# Supplementary material for: A cell-based Papain-like Protease (PLpro) activity assay for rapid detection of active SARS-CoV-2 infections and antivirals
Source: PLoS One. 2024 Dec 26;19(12):e0309305. doi: 10.1371/journal.pone.0309305 (PMC11670956; doi:10.1371/journal.pone.0309305)
Supplement: S1 File — (DOCX) [file pone.0309305.s001.docx]

**Figure 2**

| **Peptide** |  |  |  |
| --- | --- | --- | --- |
| **P4** | **PLpro nM** | **RFU** | **RFU** |
|  | 210 | 16869 | 14201 |
|  | 105 | 8283 | 7411 |
|  | 52 | 3964 | 4887 |
|  | 26.5 | 2260 | 2269 |
|  | 13.125 | 1183.8 | 1270 |
|  | 6.56 | 845 | 935 |
|  | 3.28 | 547 | 576.5 |
|  | 1.64 | 171 | 216.8 |
|  |  |  |  |
|  | **PLpro nM** | **RFU** | **RFU** |
| **Pun 16** | 100 | 1231112 | 1059964 |
|  | 25 | 1100602 | 1120336 |
|  | 6.25 | 403831 | 377247 |
|  | 1.5625 | 164943 | 69101 |
|  | 0.390625 | 29716 | 27104 |
|  | 0.097656 | 2948 | 12258 |
|  | 0.024414 | 7600 | 5886 |
|  | 0.006104 | 4559 | 4422 |
|  |  |  |  |
|  |  |  |  |
|  | **PLpro nM** | **RFU** | **RFU** |
| **Pun74** | 100 | 844253 | 872022 |
|  | 25 | 816429 | 815099 |
|  | 6.25 | 695434 | 728581 |
|  | 1.5625 | 491163 | 452478 |
|  | 0.390625 | 272118 | 273453 |
|  | 0.097656 | 263415 | 228940 |
|  | 0.024414 | 219803 | 217241 |
|  | 0.006104 | 212670 | 212907 |

Figure 3

| Sars-1 | **Time [minutes]** | |  |  |  |  |  |  |  |  |
| --- | --- | --- | --- | --- | --- | --- | --- | --- | --- | --- |
| P16 | **0** | **60** | **delta** |  | Pun16 | **0** | **60** | **delta** |  | **percent** |
|  | 59452 | 2758535 | **2699083** |  |  | 102281 | 286320 | 184039 |  |  |
|  | 60962 | 2945271 | **2884309** |  |  | 122230 | 3072881 | 2950651 |  |  |
|  | 70014 | 2825533 | **2755519** |  |  | 147044 | 3156100 | 3009056 |  |  |
| **mean** |  |  | 2779637 |  |  |  |  | 2047915 |  | 73.7 |
|  |  |  |  |  |  |  |  |  |  |  |
| Sars-2 |  |  |  |  |  |  |  |  |  |  |
| P16 | **0** | **60** | **delta** |  | Pun16 | **0** | **60** | **delta** |  | **percent** |
|  | 64044 | 2617709 | **2553665** |  |  | 38010 | 1049481 | 1011471 |  |  |
|  | 54931 | 2300049 | **2245118** |  |  | 40148 | 1144042 | 1103894 |  |  |
|  | 53910 | 2136376 | **2082466** |  |  | 45420 | 1160381 | 1114961 |  |  |
| **mean** |  |  | 2293750 |  |  |  |  | 1076775 |  | 46.9 |
|  |  |  |  |  |  |  |  |  |  |  |
|  |  |  |  |  |  |  |  |  |  |  |
| NL63 |  |  |  |  |  |  |  |  |  |  |
| P16 | **0** | **60** | **delta** |  | Pun16 | **0** | **60** | **delta** |  | **percent** |
|  | 35333 | 521332 | **485999** |  |  | 18666 | 51557 | 32891 |  |  |
|  | 18380 | 107828 | **89448** |  |  | 17779 | 56575 | 38796 |  |  |
|  | 20838 | 164339 | **143501** |  |  | 18997 | 34335 | 15338 |  |  |
| **mean** |  |  | 239649.3 |  |  |  |  | 29008.33 |  | 12.1 |
|  |  |  |  |  |  |  |  |  |  |  |
| MERS |  |  |  |  |  |  |  |  |  |  |
| P16 | **0** | **60** | **delta** |  | Pun16 | **0** | **60** | **delta** |  | **percent** |
|  | 14624 | 21250 | **6626** |  |  | 10683 | 10689 | 6 |  |  |
|  | 14212 | 21642 | **7430** |  |  | 10333 | 10381 | 48 |  |  |
|  |  |  |  |  |  |  |  | 0 |  |  |
| **mean** |  |  | 7028 |  |  |  |  | 18 |  | 0.26 |
|  |  |  |  |  |  |  |  |  |  |  |
| 229E |  |  |  |  |  |  |  |  |  |  |
| P16 | **0** | **60** | **delta** |  | Pun16 | **0** | **60** | **delta** |  | **percent** |
|  | 15581 | 26194 | **10613** |  |  | 10793 | 11704 | 911 |  |  |
|  | 13973 | 24207 | **10234** |  |  | 10659 | 10919 | 260 |  |  |
|  |  |  |  |  |  |  |  | 0 |  |  |
| **mean** |  |  | 10423.5 |  |  |  |  | 390.3 |  | 3.74 |
|  |  |  |  |  |  |  |  |  |  |  |
| OC43 |  |  |  |  |  |  |  |  |  |  |
| UbiQ | **0** | **60** | **delta** |  | Pun16 | **0** | **60** | **delta** |  | **percent** |
|  | 3723 | 90529 | **86806** |  |  | 11155 | 10918 | -237 |  |  |
|  | 3940 | 127652 | **123712** |  |  | 11982 | 11336 | -646 |  |  |
|  |  |  |  |  |  |  |  | 0 |  |  |
| **mean** |  |  | 105259 |  |  |  |  | -294.333 |  | -0.28 |
|  |  |  |  |  |  |  |  |  |  |  |
| HKU1 |  |  |  |  |  |  |  |  |  |  |
| UbiQ | **0** | **60** | **delta** |  | Pun16 | **0** | **60** | **delta** |  | **percent** |
|  | 4036 | 15423 | **11387** |  |  | 10645 | 10135 | -510 |  |  |
|  | 3310 | 13678 | **10368** |  |  | 10633 | 10399 | -234 |  |  |
|  |  |  |  |  |  |  |  | 0 |  |  |
| **mean** |  |  | 10877.5 |  |  |  |  | -248 |  | -2.28 |
|  |  |  |  |  |  |  |  |  |  |  |
| **USP18** |  |  |  |  |  |  |  |  |  |  |
| ISG15 | **0** | **60** | **delta** |  | Pun16 | **0** | **60** | **delta** |  | **percent** |
|  | 175061 | 1533598 | **1358537** |  |  | 326 | 308 | -18 |  |  |
|  | 180119 | 1482285 | **1302166** |  |  | 318 | 295 | -23 |  |  |
|  |  |  |  |  |  |  |  | 0 |  |  |
| **mean** |  |  | 1330352 |  |  |  |  | -13.7 |  | 0.00 |
|  |  |  |  |  |  |  |  |  |  |  |
| **AdenoL3** |  |  |  |  |  |  |  |  |  |  |
| P4 | **0** | **60** | **delta** |  | Pun16 |  |  | **delta** |  | **percent** |
|  |  |  | **350** |  |  |  |  | -35.1 |  |  |
|  |  |  | **341** |  |  |  |  | -38.6 |  |  |
|  |  |  |  |  |  |  |  | 0.0 |  |  |
| **mean** |  |  | 345.6 |  |  |  |  | -24.6 |  | -7.11 |

Figure 4

| **Peptide** |  |  |  |  |  |
| --- | --- | --- | --- | --- | --- |
| **Pun 74** | **Time [minutes]** | **0.1 MOI** | | **Uninfected** | |
|  | 0 | 8602 | 5656 | 8602 | 5656 |
|  | 15 | 67365 | 67416 | 8491 | 8644 |
|  | 30 | 99208 | 99683 | 11222 | 11512 |
|  | 60 | 135314 | 131856 | 15810 | 16419 |
|  |  |  |  |  |  |
| **DEVD** | **Time [minutes]** | **0.1 MOI** | | **Uninfected** | |
|  | 0 | 67749 | 115705 | 45719 | 50896 |
|  | 15 | 289822 | 420138 | 161782 | 168777 |
|  | 30 | 594094 | 621383 | 333369 | 347373 |
|  | 60 | 1343884 | 1378115 | 745609 | 786504 |

Figure 5

| Time |  | MOI | | | | | |
| --- | --- | --- | --- | --- | --- | --- | --- |
| 2h |  | 0.1 | | 0.01 | | 0 | |
|  | 0 | 10200 | 5977 | 10200 | 5977 | 10200 | 5977 |
|  | 5 | 7431 | 7780 | 6403 | 6037 | 10200 | 5977 |
|  | 15 | 21076 | 22478 | 13361 | 12793 | 21893 | 12031 |
|  | 30 | 34872 | 40467 | 20344 | 19828 | 30940 | 18342 |
|  | 45 | 45833 | 51228 | 26028 | 25715 | 36478 | 23653 |
|  | 60 | 54267 | 58796 | 30843 | 30933 | 40166 | 28166 |
|  |  |  |  |  |  |  |  |
| 6h |  | 0.1 | | 0.01 | | 0 | |
|  | 0 | 5990 | 5721 | 5990 | 5721 | 5990 | 5721 |
|  | 5 | 8422 | 8442 | 5265 | 4940 | 5990 | 5721 |
|  | 15 | 30233 | 30657 | 11499 | 10265 | 12998 | 12060 |
|  | 30 | 48081 | 47556 | 18194 | 15952 | 20908 | 19147 |
|  | 45 | 61890 | 59870 | 24959 | 20630 | 28156 | 25224 |
|  | 60 | 71925 | 68158 | 29175 | 24706 | 34414 | 30462 |
|  |  |  |  |  |  |  |  |
|  |  |  |  |  |  |  |  |
| 8h |  | 0.1 | | 0.01 | | 0 | |
|  | 0 | 9290 | 7120 | 9290 | 7120 | 9290 | 7120 |
|  | 5 | 11237 | 11666 | 8347 | 6046 | 9290 | 7120 |
|  | 15 | 47426 | 49793 | 23598 | 16530 | 15917 | 15915 |
|  | 30 | 75715 | 74954 | 34326 | 26488 | 22654 | 21573 |
|  | 45 | 95767 | 92404 | 45999 | 32824 | 29383 | 27412 |
|  | 60 | 111472 | 103906 | 51926 | 38817 | 34399 | 31975 |
|  |  |  |  |  |  |  |  |
| 24h |  |  |  |  |  |  |  |
|  |  | 0.1 | | 0.01 | | 0 | |
|  | 0 | 5667 | 5638 | 5667 | 5638 | 5667 | 5638 |
|  | 5 | 14569 | 14707 | 14397 | 23915 | 5667 | 5638 |
|  | 15 | 74145 | 72590 | 75484 | 114399 | 11734 | 12051 |
|  | 30 | 112471 | 106932 | 111103 | 157426 | 17754 | 18451 |
|  | 45 | 136087 | 129634 | 131436 | 177661 | 22514 | 24094 |
|  | 60 | 149276 | 139015 | 143001 | 170922 | 26698 | 28805 |

Figure 6

|  | **GRL0617 [uM]** | | | | | |
| --- | --- | --- | --- | --- | --- | --- |
| **Time [min]** | **100 uM** | | **12.5 uM** | | **3.125 uM** | |
|  | **RFU** | | **RFU** | | **RFU** | |
| **0** | 7051 | 6517 | 7051 | 6517 | 7051 | 6517 |
| **15** | 17061 | 16931 | 68837 | 66859 | 75530 | 77060 |
| **30** | 24262 | 23806 | 95641 | 92549 | 105495 | 107085 |
|  |  |  |  |  |  |  |
|  |  |  |  |  |  |  |
|  | **medium + virus** | | **medium + DMSO**  **no virus** | | **medium only** | |
|  | **RFU** | | **RFU** | | **RFU** | |
| **0** | 7051 | 6517 | 7051 | 6517 | 7051 | 6517 |
| **15** | 80960 | 74295 | 13594 | 12365 | 10988 | 10893 |
| **30** | 111065 | 104671 | 16904 | 17701 | 15209 | 15117 |

Figure 7

| **Time [minutes]** | Serum 1:40 +virus | |
| --- | --- | --- |
|  | **RFU** | |
| 0 | 5960 | 5673 |
| 15 | 9390 | 9221 |
| 30 | 12475 | 12682 |
|  |  |  |
|  | Serum 1:160 +virus | |
|  | **RFU** | |
| 0 | 5442 | 5241 |
| 15 | 8365 | 7943 |
| 30 | 11272 | 10656 |
|  |  |  |
|  | Serum 1:640 +virus | |
|  | **RFU** | |
| 0 | 5726 | 5376 |
| 15 | 8726 | 8430 |
| 30 | 11916 | 11208 |
|  |  |  |
|  | Serum 1:2560 +virus | |
|  | **RFU** | |
| 0 | 8596 | 9371 |
| 15 | 25778 | 27258 |
| 30 | 37571 | 40259 |
|  |  |  |
|  | Medium +virus | |
|  | **RFU** | |
| 0 | 10525 | 11565 |
| 15 | 37715 | 39407 |
| 30 | 56451 | 56583 |
|  |  |  |
|  | Medium | |
|  | **RFU** | |
| 0 | 6748 | 5003 |
| 15 | 8249 | 7804 |
| 30 | 11089 | 10305 |

Figure 9

| PLPro | **Minutes** | **A** | | **B** | | **C** | |
| --- | --- | --- | --- | --- | --- | --- | --- |
|  |  | **RFU** | | **RFU** | | **RFU** | |
|  | 0 | 139 | 134.6 | 142 | 144.8 | 126.6 | 126.5 |
|  | 30 | 150.2 | 144.5 | 152.3 | 148.1 | 422.3 | 426.1 |
|  | 60 | 147.1 | 143.7 | 144.5 | 143.2 | 970 | 974.3 |
|  |  |  |  |  |  |  |  |
|  |  |  |  |  |  |  |  |
|  |  |  |  |  |  |  |  |
| DEVD | **Minutes** | **A** | | **B** | | **C** | |
|  |  | **RFU** | | **RFU** | | **RFU** | |
|  | 0 | 40.25 | 43.46 | 40.51 | 41.55 | 77.24 | 75.4 |
|  | 30 | 53.59 | 54.85 | 41.88 | 41.82 | 141.5 | 140.8 |
|  | 60 | 71.33 | 72.35 | 46.94 | 44.23 | 218.1 | 217.8 |

Figure 10

| **ID** | **Mpro** | **PLPro** |
| --- | --- | --- |
| 1 | 72,128 | 760,510 |
| 3 | 56,135 | 351,941 |
| 4 | 269,173 | 1,862,801 |
| 6 | 20,169 | 74,694 |
| 7 | 1,389 | 616,779 |
| 8 | 27,261 | 1,343,877 |
| 9 | 8,317 | 81,256 |
| 10 | 3,282 | 151,558 |
| 11 | 6,717 | 12,037 |
| 12 | 42,509 | 1,694,917 |
| 13 | INV | INV |
| 14a | 44,856 | 823,884 |
| 14b | 189,507 | 1,939,194 |
| 15 | 183,788 | 3,768,399 |
| 16 | 19,103 | 534,755 |
| 17 | 26,651 | 752,027 |
| 18 | 203,135 | 2,749,004 |
| 19a | 17,596 | 87,800 |
| 19b | INV | INV |
| 20 | 275,902 | 1,091,985 |
| 21a | 321,324 | 3,692,954 |
| 21b | 364,133 | 3,740,001 |
| 22 | 62,769 | 761,528 |

Figure 11

| **ID** |  | **Ratio N1/RnaseP** | | | | |
| --- | --- | --- | --- | --- | --- | --- |
| **1** |  | 20 | 44 |  |  |  |
| **3** |  | 11 | 58 |  |  |  |
| **4** |  | 266 | 115 |  |  |  |
| **6** |  | 0 | 0 |  |  |  |
| **7** |  | 24 | 19 |  |  |  |
| **8** |  | 145 | 63 |  |  |  |
| **9** |  | 160 | 113 |  |  |  |
| **10** |  | 18 | 4 |  |  |  |
| **11** |  | 0 | 7 |  |  |  |
| **12** |  | 50 | 58 |  |  |  |
| **13** |  | 353 | 64 |  |  |  |
| **14a** |  | 0 | 0 |  |  |  |
| **14b** |  | 19 | 16 |  |  |  |
| **15** |  | 38 | 9 | 28 | 171 | 95 |
| **16** |  | 89 | 63 | 80 | 198 | 46 |
| **17** |  | 0 | 72 | 0 | 34 |  |
| **18** |  | 111 | 149 | 174 |  |  |
| **19a** |  | 80 | 104 |  |  |  |
| **19b** |  | 82 |  |  |  |  |
| **20** |  | 22 | 41 |  |  |  |
| **21a** |  | 23 | 2 | 8 |  |  |
| **21b** |  | 20 |  |  |  |  |
| **22** |  | 12 | 92 | 146 |  |  |

Figure 12

|  | **Ratio PLPro [RFU]** | |
| --- | --- | --- |
| **ID** | **Neg** | **Pos** |
| 1 |  | 87.6 |
| 3 |  | 47.6 |
| 4 |  | 97 |
| 6 | 16.1 |  |
| 7 |  | 52.1 |
| 8 |  | 88.2 |
| 9 |  | 15.3 |
| 10 | 11.5 |  |
| 11 | 1.5 |  |
| 12a |  | 112.7 |
| 14 |  | 63.4 |
| 15 |  | 151.1 |
| 16 |  | 38.7 |
| 17 |  | 33.7 |
| 18 |  | 116 |
| 19a |  | 8.7 |
| 20 |  | 56.8 |
| 21a |  | 179.3 |
| 22 |  | 36.8 |

Table 3

| **ID** | **QV** | | | | **MPro** | | | | **PLPro** | | | |
| --- | --- | --- | --- | --- | --- | --- | --- | --- | --- | --- | --- | --- |
| **1** | 1,083,968 | 891,466 |  |  | 72,128 |  |  |  | 969,612 | 760,510 |  |  |
| **3** | 811,249 | 667,578 |  |  | 54,768 | 57,501 |  |  | 348,844 | 355,038 |  |  |
| **4** | 1,028,477 | 421,010 | 892,693 | 573,648 | 251,163 | 18,010 | 33,045 |  | 1,223,055 | 251,348 | 639,746 | 537,777 |
| **6** | 464,927 | 457,213 | 466,089 |  | 28,283 | 12,054 |  |  | 64,881 | 26,883 | 132,319 | 74,694 |
| **7** | 1,521,995 | 846,302 |  |  | 1,389 |  |  |  | 769,511 | 464,047 | 616,799 |  |
| **8** | 1,513,501 | 1,532,937 |  |  | 27,261 |  |  |  | 2,070,109 | 617,644 |  |  |
| **9** | 370,135 | 259,985 | 881,344 | 612,014 | 8,690 | 7,943 |  |  | 85,877 | 82,341 | 76,960 | 79,846 |
| **10** | 281,286 | 267,162 | 2,620,550 |  | 3,282 | 28,180 |  |  | 137,198 | 221,474 | 5,146 |  |
| **11** | 451,640 | 368,503 | 2,105,355 | 195,628 | 2,506 | 3,239 | 16,217 | 4,905 | 17,519 | 14,510 | 9,350 | 6,770 |
| **12** | 1,591,245 | 1,603,143 | 1,318,548 |  | 42,686 | 26,144 | 58,698 |  | 3,057,520 | 1,062,480 | 964,750 |  |
| **13** | 918,869 | 716,034 |  |  | 8,864 | 5,116 |  |  | 182,486 | 79,146 |  |  |
| **14a** | 1,318,313 | 1,401,097 | 1,181,912 | 1,300,440 | 34,750 | 42,293 | 57,524 | 44,855 | 855,971 | 1,022,358 | 593,323 | 823,884 |
| **14b** | 1,764,610 | 1,581,024 | 1,405,597 |  | 164,217 | 81,480 | 322,823 |  | 2,254,558 | 1,976,663 | 1,586,361 |  |
| **15** | 2,705,418 | 2,641,915 | 2,347,991 | 2,278,797 | 173,129 | 192,066 | 94,868 | 275,090 | 3,768,661 | 4,533,563 | 3,805,511 | 2,965,859 |
| **16** | 1,494,472 | 1,486,689 | 1,159,788 |  | 19,889 | 10,144 | 27,276 |  | 657,595 | 569,307 | 377,362 |  |
| **17** | 2,475,337 | 1,974,267 | 2,242,032 |  | 24,942 | 43,636 | 11,375 |  | 922,489 | 642,575 | 691,016 |  |
| **18** | 2,501,893 | 2,238,745 |  |  | 97,179 | 309,091 |  |  | 3,756,252 | 1,741,756 |  |  |
| **19a** | 1,033,529 | 856,936 | 1,284,606 | 884,094 | 16,867 | 13,414 | 28,573 | 11,529 | 79,281 | 53,628 | 155,850 | 62,440 |
| **19b** | 153,585 | 129,370 | 129,768 |  | 5,461 | 5,695 | 2,529 |  | -30,498 | -105,007 | -214,001 |  |
| **20** | 2,078,275 | 1,972,247 | 1,715,449 |  | 323,934 | 264,044 | 239,728 |  | 1,431,523 | 1,055,036 | 789,395 |  |
| **21a** | 2,309,591 | 1,985,548 | 1,884,774 |  | 390,389 | 351,867 | 221,717 |  | 5,495,992 | 4,191,376 | 1,391,495 |  |
| **21b** | 2,403,001 | 2,189,436 |  |  | 452,601 | 275,664 |  |  | 5,028,446 | 2,451,555 |  |  |
| **22** | 2,014,060 | 2,172,944 | 2,013,586 |  | 78,505 | 68,385 | 40,408 |  | 726,073 | 832,439 | 726,073 |  |

SI FIG1

| **A** | **Lysate [ul]** | **Positive** | | | **Negative** | | |
| --- | --- | --- | --- | --- | --- | --- | --- |
|  | 100 | 149078 | 150482 | 143874 | 31123 | 45365 | 45184 |
|  | 50 | 44829 | 53695 | 53294 | 25448 | 24305 | 21242 |
|  | 25 | 16744 | 31135 | 37539 | 9509 | 7904 | 12236 |
|  | 12.5 | 13639 | 10917 | 12610 | 6811 | 4676 | 7361 |
|  | 6.25 | 4761 | 4873 | 4197 | 3072 | 4915 | 6160 |
|  | 3.125 | 3609 | 2815 | 2232 | 2185 | 1604 | 3863 |
|  | 0 | 1362 | 891 | 629 | 301 | 3181 | 272 |
|  |  |  |  |  |  |  |  |
| **B** | **Lysate [ul]** | **Positive** | | | **Negative** | | |
|  | 100 | 804428 | 976243 | 966638 | 202742 | 177471 | 168112 |
|  | 50 | 360278 | 352421 | 295645 | 87592 | 111741 | 117960 |
|  | 25 | 155321 | 99339 | 184345 | 31058 | 15514 | 47206 |
|  | 12.5 | 97297 | 63415 | 65126 | -55590 | -11203 | -27059 |
|  | 6.25 | -17033 | 14730 | 3233 | -115587 | -94658 | -139277 |
|  | 3.125 | -28090 | -6466 | -10684 | -112083 | -59000 | -53609 |
|  | 0 | -44325 | -29433 | -42446 | -90396 | -42494 | -190405 |
|  |  |  |  |  |  |  |  |
|  |  |  |  |  |  |  |  |
|  | **Lysate [ul]** | **Positive** | | | **Negative** | | |
| **C** | 100 | 857303 | 997229 | 993701 |  | 1011320 | 1396999 |
|  | 50 |  | 469322 | 516727 | 640267 | 734734 | 558427 |
|  | 25 | 362261 | 270020 | 447634 | 486455 | 363059 | 385268 |
|  | 12.5 | 212885 | 207596 | 230592 | 275752 | 470014 | 274950 |
|  | 6.25 | 153469 | 198268 | 148282 | 180877 | 178490 | 226245 |
|  | 3.125 | 110486 | 117227 | 115428 | 164199 | 145817 | 155815 |
|  | 0 | 63994 | 61213 | 62272 | 65665 | 66293 | 68573 |

SI FIG 2

| **Virus** |  | **ISG15** | | **ACE2 peptide** | |
| --- | --- | --- | --- | --- | --- |
|  |  | **RFU** | | **RFU** | |
| 229E | infected | 1054 | 732 | 40946 | 42203 |
|  | not infected | 512 | 535 | 38170 | 38442 |
|  |  |  |  |  |  |
|  |  | **RFU** | | **RFU** | |
| OC43 | infected | 1643 | 1832 | 5479 | 5385 |
|  | not infected | 1017 | 1068 | 5403 | 5301 |
|  |  |  |  |  |  |
|  |  | **RFU** | | **RFU** | |
| NL63 | infected | 1893 | 1883 | 4469 | 4309 |
|  | not infected | 1438 | 1370 | 4688 | 4589 |
